# Understanding the social and structural context of oral PrEP delivery: an ethnography exploring barriers and facilitators impacting transgender women who engage in street-based sex work in Baltimore, Maryland

**DOI:** 10.1186/s12981-023-00556-6

**Published:** 2023-09-20

**Authors:** Erin E. Cooney, Katherine H. A. Footer, Jennifer L. Glick, Anna Passaniti, Meridian Howes, Susan G. Sherman

**Affiliations:** 1 Department of International Health, Johns Hopkins Bloomberg School of Public Health, Baltimore, USA; 2 Department of Health, Behavior and Society, Johns Hopkins Bloomberg School of Public Health, Baltimore, USA

**Keywords:** Transgender women, Sex work, Preexposure prophylaxis, HIV, Risk environment

## Abstract

Transgender women who sell sex (TWSS) experience high rates of HIV acquisition. Antiretrovirals for pre-exposure prophylaxis (PrEP) represent an efficacious HIV prevention strategy. The social and structural factors affecting PrEP delivery amongst TWSS are underexplored in the literature. We conducted ethnographic research to examine how multilevel social and structural factors manifest in TWSS’s lived experiences and affect PrEP delivery and use. Twenty-four transgender women were recruited from the SAPPHIRE cohort and completed interviews focused on barriers and facilitators to PrEP engagement in the context of street-based sex work. Stakeholder interviews (N = 7) were also conducted. Our findings suggest there are unique features of the risk environment that can collectively impede PrEP use among TWSS.

## Background

Pre-exposure prophylaxis (PrEP) represents a highly efficacious HIV prevention strategy when adherence is high [21]. Further, PrEP provides those who experience elevated HIV risk increased autonomy compared to other prevention strategies such as condoms [2]. Transgender women (defined as people who identify as women and were assigned a male sex at birth) experience disproportionately elevated HIV incidence rates and can benefit from PrEP. A 2019 systematic review and meta-analysis [5] found an estimated 14% of transgender women in the USA are living with HIV. There are disparities by race and ethnicity, with an estimated prevalence of 44%, 26% and 7% among Black/African American, Hispanic/Latina and White transgender women, respectively [5]. Recent HIV surveillance data for Baltimore City indicates that HIV prevalence is 2% in the general population, while studies have found an HIV prevalence of 56% among Black and Latina transgender women and 40% among transgender women who sell sex (TWSS) in Baltimore [30, 38, 40] highlighting unmet HIV prevention needs.

As a result of structural marginalization and societal exclusion in terms of education and employment, an estimated 24–75% of transgender women in the United States, particularly Black and Latina transgender women, report engagement in sex work at some point in their lives [5]. Engagement in sex work can further elevate social and structural vulnerabilities, thereby increasing vulnerability to HIV [38]. TWSS experience a disproportionate burden of HIV compared to sex workers of other genders, with HIV prevalence of 27.3% among TWSS globally, compared to 11.8% among cisgender female sex workers (CFSW) and 15.1% among cisgender male sex workers [3, 4, 25].

A growing body of literature has focused on identifying factors impacting PrEP continuum engagement amongst transgender populations [30, 37, 46]. Findings include: concerns around medication side effects, in particular interactions with hormones [17], health provider distrust due to enacted and anticipated transphobia [10, 35], PrEP stigma and its intersection with HIV stigma [6, 11], exclusion of trans people in PrEP marketing [35], structural instability due to economic marginalization and homelessness [46]. Conversely, potential facilitators to PrEP engagement include individual self-perceived HIV risk [9], interpersonal peer influence and social support and access to gender affirming PrEP service sites [20]. In Baltimore City, despite efforts by the Health Department to prioritize PrEP awareness and uptake among identified priority populations (including transgender women) through social innovation projects, recent studies indicate important PrEP continuum gaps and suboptimal PrEP uptake. For example, among Black and Latina transgender women in Baltimore and nearby Washington, DC, PrEP acceptance was high (75%) despite low uptake (17%) [30].

Public health discourse has increasingly focused on social and structural factors that influence HIV-related risk-taking and risk-reduction among marginalized populations [3, 34, 39]. For instance, criminalized environments can constrain individual choice and agency around HIV harm reduction strategies (e.g., condom use, uptake of health care services) [1, 13, 28]. Rhodes’ risk environment positions individual HIV risk and the enabling steps necessary for its reduction (e.g., PrEP uptake) within “a space, whether social or physical where a variety of environmental factors interact to increase the chances of risk occurring” (pg. 193) [33]. Rhodes’ framework focuses on physical, economic, social and policy environmental factors that can contribute to success of failure of HIV intervention strategies, as opposed to biologically-determined or individualistic approaches, which fail to account for individuals’ environments [32]. Our study aims to explore how social context and environment may influence PrEP acceptability, uptake, and adherence among TWSS in Baltimore City, to inform community-engaged intervention development.

## Methods

### Context and study setting

Black transgender women who are engaged in sex work have historically worked in a specific area of Baltimore City, known as the “Charles Stroll” or “The Stroll”. Despite frequent violence on The Stroll, including the unsolved homicides of multiple Black transgender women over the last decade, it has remained a central work venue for TWSS and as a physical space associated with community and social support for many.

### Participants and procedures

#### In-depth interviews with TWSS

TWSS (N = 24) were recruited from a cohort study with transgender and cisgender women engaged in street-based sex work in Baltimore City, The SAPPHIRE Study [38]. TWSS participated in the present qualitative sub-study between June 2017 and March 2018. Qualitative interviews explored lived sex work experiences and barriers and facilitators to PrEP engagement. The guide was piloted with a research assistant, a Black transgender woman who had worked on The Stroll, to refine language and terms and ensure questions were relevant and well-understood by the study population. TWSS were purposively sampled to ensure diversity of age and length of engagement in street-based sex work. Although there were no explicit inclusion criteria based on race or ethnicity, all participants identified as Black. Interviews took place in private, safe, and convenient locations, including local LGBTQ community organizations and research offices. Interviews lasted approximately 60 min and were audio recorded and transcribed verbatim. All participants were compensated with a $40 Visa gift card. Referrals for health and social services (e.g., STI testing, pro bono legal support, nutrition services) were offered where appropriate.

#### Stakeholder interviews

Seven stakeholder interviews lasting approximately 50 min were completed with local stakeholders including those who provide clinical and support services to transgender women. Interviewees were selected based on the senior author’s knowledge and past ethnographic work in the community, with snowball sampling based on stakeholder recommendations. All interviews were conducted in a private space at the participants’ offices. Interviews focused on understanding key social and structural vulnerabilities facing the transgender community as they related to PrEP engagement. Interviews were audio recorded and transcribed verbatim. No compensation was provided.

The co-first author (KHAF) led data collection with support from co-authors (EC, JG, AP, MH) all of whom are trained in qualitative research and analysis. All procedures were approved by the [REDACTED] Institutional Review Board.

### Analysis

Ethnographic data were analyzed adapting a pluralistic theory and data driven approach that was both inductive and deductive [22]. The analysis team (KF, EC, AP, MH) developed a codebook based on topical and thematic codes derived from the interview guides and inductive codes derived from open coding. The initial codebook was applied to 3 interview transcripts and revised iteratively by the coding team. Three team members (EC, AP, MH) coded interview transcripts. To ensure rigor, regular team meetings allowed for a close examination of any coding inconsistencies. A subset of six transcripts were double-coded to assess consistency in code application. Transcripts were fully de-identified. With participant approval, TWSS participants were assigned a pseudonym to accompany quotes. Stakeholders were not asked to provide a pseudonym at the time of the interview and therefore were assigned a participant number (e.g., Stakeholder 1).

An adapted interview codebook was used for stakeholder interviews. The first authors then used a framework analysis that drew upon Rhodes’ “Risk Environment” framework to move from first level to second level coding [14, 33].

## Results

All TWSS study participants identified as Black with 25% identifying as multiracial (i.e., Black and another race); participants ranged in age from 18 to 58 years old (mean = 34). All participants reported having heard of PrEP while 17% reported having ever taken PrEP (Table 1). Findings are presented below according to Rhodes’ risk environment domains.

**Table 1:** Demographic characteristics of the 24 transgender women who sell sex work who were interviewed for this study

N	24
Age (mean, range)	34 (18–58)
Race	
Black only (%)	75%
Multiracial [Black and one or more other race(s)] (%)	25%
Time since initial entry into sex work, years (mean, range)	15 (0–32)
Heard of PrEP (%)	100%
Ever taken PrEP (%)	17%

### Relationship with sex work and HIV risk perceptions

All TWSS and stakeholder interviews described sex work as an occupation that many Black trans women will engage in at some point across the life course due to an oppressive social context, which greatly limits access to other forms of employment. As one participant explained:

*I think when you deny people human rights, you put them in situations where they feel like it’s absolutely necessary for them to circumvent the law just to survive everyday life. I don’t believe that half of the transwomen that I have become very close with would have ever become sex workers if they could have found fair employment and things of that nature. I don’t think it would have ever been something that they would have been enticed by.* (Michelle, age 43)

Multiple participants described their introduction to The Stroll as exciting and a form of societal debut, but this contrasted strongly with descriptions of remaining on The Stroll over a sustained period. As one participant described:

*Honey, a typical night was pumping down the street in your heels—oh my God—sitting out there, waiting for a date, being disrespected. Boys coming up offering you $10 and $5. Oh my God—being chased up and down the street from the police on nights that they wanted to lock you up. We had to hide in the alley or under a car. Girl, it was a mess. It was cute, back in the day. Back in the day it was fun, but now it’s not fun anymore because now the boys are attacking, I just lost a friend the other day who got killed and shot and burned in the house.* (Dana, age 30)

Participants shared that they understood that their chance of HIV infection was most acute while engaged in sex work. As this participant explained:

*I think anybody—cis women, trans women—anybody should consider PrEP. But as far as trans women working on The Stroll—the girls on The Stroll—I feel it should be considered more often, because you're trying to prevent HIV. So that's one place where I feel you can push towards them being on PrEP.* (Bailey, age 19)

### Policy environment surrounding PrEP delivery

Interviews with stakeholders indicated that alongside complicated PrEP insurance policies, barriers to legal identity documents that match gender identity could hamper willingness and/or ability to access healthcare, and therefore PrEP uptake.

*Sometimes there are barriers to PrEP access in terms of simply going to a pharmacist and having to give legal names…* (Stakeholder 6)

One participant who was aware of PrEP had rejected PrEP services because of state level laws prohibiting PrEP distribution to minors without parental consent at the time of the interview. Stakeholder and TWSS interviews indicated that girls below the age of eighteen are regularly on The Stroll.

*My friend was interested in getting on PrEP and we both went to go get tested, and start PrEP. But when I went, I was like 17, so I needed like my parents' consent on that. And I really don't, you know, talk to my parents. *(Nikki, age 18)

### Economic environment surrounding PrEP delivery

Sex work was described by both TWSS and stakeholders as a way to counter economic vulnerability generated by a transphobic and discriminatory social context.

*People do it [sex work] because they have to do it, because they can’t apply for jobs, or they don’t have good education, or they never graduated from school, I’m just telling you reasons why girls haven’t built a career.* (Brianna, age 39)

Participants also described how economic need generates HIV risk-taking on The Stroll, while simultaneously leaving little or no room to contemplate healthcare engagement.

*If this person offers you $40 to do oral with a condom, but this person right here is offering $80 to do it without the condom… I mean, you're going to take both, but you're going to mainly go for that $80 without the condom. *(Brooklyn, age 35)

*When I was out there, I knew that I was at risk, but I didn't want to talk to a doctor about anything – because if I get some bad news, that's going to stop my money flow. I’m going to be depressed, I'm not going to make no money, and I'm not going to be able to move forward.* [Stakeholder 1 (former sex worker)]

Stakeholders shared that there was an active informal economy around prescription drugs, including PrEP, among TWSS, with negative implications for downstream adherence.

*PrEP is the hot thing right now. People are getting PrEP prescriptions and selling it to people. But then my other concerns are just like, are people getting the right information to use it in a way that it can actually work for them? I've heard that some people are like using it just when they're having potential risky exposure.* (Stakeholder 7)

### Social environment surrounding PrEP delivery

Most participants experienced sex work stigma. This was evident in the way participants talked about their own and others’ engagement in sex work.

Definitely relevant [PrEP for Trans women]. These are whores, mainly. So, when you think of a whore, you want to protect a whore, you know whores are nasty. I guess that includes myself. (Zoe, age 24)

Stakeholder interviews indicated that many TWSS are likely uncomfortable accessing healthcare due to concerns around experiencing sex work discrimination, subsequently limiting PrEP uptake opportunities.

*You can walk into a health clinic and just get this vibe that sex work is dirty and nasty and you shouldn't be doing it, and somehow the people there seem to frame it that way.* (Stakeholder 5)

Stakeholder and participants spoke of TWSS obtaining PrEP from within their social circles, and the problems with adherence that indirectly arise from trans women obtaining PrEP outside health care settings.

*I know some people that be like, ‘Girl, you have any more PrEP pills over there? I need some.’ It's stuff like that, not knowing you're supposed to take it every freaking day. I will just say this, it's a lot of girls on The Stroll that have not had any guidance in life. They go to other girls on The Stroll. They go to other peers that's in their same predicament for resources. They don't really know what they're doing.* (Chanel, age 33)

Another aspect of the social environment, illicit drug use, was described by some TWSS as destabilizing and incompatible with PrEP uptake and adherence.

*I was having unprotected sex very often. All I was focusing on was the next drug.*

*It [daily oral PrEP] probably would’ve been kicked to the curb. *(Layleen, age 48)

Many participant narratives indicated that PrEP advertising contributed to stereotypes of promiscuity within the LGBTQ community, singling out gay men and transgender groups and limiting PrEP acceptance.

*When you see PrEP, even commercials on TV, who do you see? Homosexual people. You don’t see cis people on like the commercial or flyer. *(Bee, age 37)

TWSS and stakeholders pointed to HIV stigma, and the conflation of antiretrovirals for HIV treatment with PrEP for HIV prevention.

*Me taking a pill [PrEP], somebody's going to be like, are you HIV positive? Because people, especially in this lower income society, are like, you must have HIV since you're taking Truvada. (Alexa, age 18)*

*You have to get tested for HIV before starting PrEP and people still have a stigma around wanting to go get tested. That can be a whole thing with people too. *(Stakeholder 7)

More broadly, some TWSS and stakeholders talked of medical conspiracy theories around the emergence of PrEP, impacting PrEP acceptance and uptake.

They [transgender women] know what PrEP is, they just don't want to take it. For all they know, it could be a pill to kill you. We never really know what it is. It could be infecting our body. The doctor just says, hey, take PrEP. (Kiki, age 25)

*We asked people [transgender women] if there was a pill that you could take once a day to prevent getting HIV, would you take it? And something like 87% of the people that we met on the street said yes. And the ones that said no had very specific reasons that were all focused on- that's not real, it's a hoax. It's a government experiment on Black people again. It was all really couched in this sort of medical mistrust. And the sense that there was something in that pill that wasn't really about preventing HIV but was about killing Black people. *(Stakeholder 5)

### Physical environment surrounding PrEP delivery

Police harassment was identified as negatively impacting outreach activities on The Stroll, which was a primary method for linking TWSS to PrEP.

*We [outreach staff] made sure that we branded ourselves in a way that we were identifiable…*

*so that the police would reduce their harassment, which they didn’t…* (Stakeholder 5)

One stakeholder reported hearing that police confiscate Truvada, based on the person's gender identity and assuming they are doing sex work. Participants additionally spoke of the physical environment of The Stroll being a setting of extreme violence, with one participant describing it as a “war zone”.

*You get shot by people that do not like trans people. You get robbed by people that might like trans people, but they just want your money. Dates get crazy and don't want to pay you all your money. You get stranded somewhere because a date takes you so far from the place. My biggest fear was always 1 day just going missing. I was scared that 1 day a man was just going to take me far out and not bring me back.* (Alexa, age 18)

This trauma-laden reality was described by a few TWSS and stakeholders as incompatible with harm reduction strategies like PrEP.

*I know quite a few people that don’t give a damn, that are going to do what they’re doing. If it happens, it happens [HIV]. There is trauma. And they’re just going to ball out till they fall out.* (Stakeholder 7)

### Structural interventions to facilitate PrEP delivery among TWSS

Legal services, including expungement and identity document services were identified by stakeholders as providing an important bridge to healthcare access, including PrEP. Many participants and stakeholders also spoke of PrEP integration into gender affirming healthcare services as critical to adherence.

*Any provider who wants to make PrEP available for trans women needs to also be their hormone provider. If you did those two things at once, they would come in and get it, they would come in for the 3 month follow-up. *(Stakeholder 5)

Many participants and stakeholders identified affordable housing as an important macro policy issue that could facilitate a more enabling environment for PrEP adherence, enhancing stability in trans women’s daily lives, accompanied by structural interventions to increase job training and opportunities. Building stronger peer interventions and using social network approaches to improve PrEP acceptance was a popular recommendation.

*A lot of people really don’t know about PrEP, but if you had navigators or peer facilitators—other trans women going to talk to other trans women, because to a certain extent we understand one another, and they would listen. *(Johana, age 48)

Another suggestion was utilizing overlapping transgender social spaces to promote PrEP, such as the Ballroom Scene, in which younger women participate, and online spaces like Facebook and Instagram. Most participants felt that any future awareness raising campaigns should be inclusive of all gender identities, rather than singling out transgender people.

*It's a pill for everybody. Everybody should be on it. It's for everyone. No matter what race, whatever. I think it should just be for everybody: cis people, transgender women, trans men, just everybody.* (Nikki, age 18)

## Discussion

Applying the risk environment framework [32], our findings provide insights into how the social and structural sex work environment can impact TWSS’s motivations, decisions, and behaviors regarding PrEP awareness, acceptance, uptake and adherence. Our findings suggest that there are unique environmental features which impact HIV-risk and uptake of PrEP including: a micro-policy environment heavily focused on condom-distribution; a micro-economic environment that negates self-care and harm reduction; a social environment of deeply embedded sex work stigma; and a physical environment of over-policing and violence. These multi-level environments synergistically impede PrEP success. Our findings confirm the syndemic [44] impact of broader socio-structural risk factors that have been identified as important to the PrEP care continuum among transgender women, including not having gender affirming documentation, deeply embedded institutional and medical mistrust among transgender and Black communities, and PrEP stigma [41, 45, 47]. Finally, TWSS and stakeholders identified enabling distal (e.g. stable housing, employment opportunities, criminal record expungement, and access to legal name change services) and proximal (e.g. integrating PrEP into gender affirming care, inclusive PrEP campaigning, and targeted peer navigation) factors that could be leveraged in intervention development to improve PrEP continuum outcomes among TWSS.

Data highlighted that felt stigma–internalized feelings of blame and shame–coupled with fear of being discriminated against [7] is associated with sex work engagement. Our findings do suggest that during acute periods of structural vulnerability (e.g., economic hardship), engagement in street-based sex work differentiates and enhances a transgender woman’s HIV risk and presents unique social and structural barriers to PrEP engagement and retention that warrant separate elucidation. Both the public health evidence [26, 29, 38] and participant descriptions suggest that TWSS are a distinct HIV at-risk group compared to their non-sex work engaged peers, PrEP is an important HIV prevention intervention.

The micro policy on The Stroll was heavily focused on condom distribution. To ensure PrEP awareness and acceptance among TWSS, targeted efforts are required to ensure PrEP promotion, particularly extending promotion responsibility from local community-based organizations to municipal health departments. Broader policy barriers relevant to all transgender women who sell sex included problems navigating insurance coverage and lack of gender affirming documentation that could have unexpected downstream implications for trans women’s structural vulnerability and PrEP adherence. Participants and key informants were keen to address the need for macro policies to combat housing shortages and address the downstream micro vulnerabilities (i.e., homelessness, housing instability) that can interfere with PrEP adherence. Consistent with broader literature [31], co-locating PrEP with gender affirming care services was identified in key informant interviews as an important way to take account of trans women’s primary health concerns (eg, gender-affirmative care) while building acceptance and trust around the delivery of HIV prevention services. Our findings suggest an increasingly enabling environment for integrated HIV prevention, with more transgender women receiving gender affirming care. Ensuring that integration of PrEP delivery with gender affirming healthcare is a cornerstone of local-level health policy should be considered by local health departments. An important policy change that has taken effect in the State of Maryland has been passage of a law allowing prescription of PrEP to minors without parental consent [24]. Given that key informants and participants consistently highlighted younger girls as the most active on The Stroll, this policy change provides a powerful example of how changes to the legal/policy environment can promote an enabling environment for PrEP success.

Consistent with broader literature [23, 29], acute economic vulnerability was the most cited reason for spending prolonged periods on The Stroll. The economic environmental risk factors on The Stroll, not only increase HIV vulnerability (e.g., condom-less sex with clients for more money), but the economic pressures and fast turnover on The Stroll impeded health-seeking behaviors, including PrEP uptake. At the macro-level, economic investment and improvements in gender affirming care resulted in a shift to receiving this care in formal, regulated healthcare settings, rather than unregulated hormones from unregulated sources. However, there seemed to be a disparity in healthcare access among transgender women currently more active on The Stroll. This was starkly illustrated by participant reports that PrEP sharing had occurred, with implications for poor or ineffective levels of adherence among trans women on The Stroll (triangulated with stakeholder interviews). Identifying this informal PrEP economy suggests there is a gap in healthcare access for some TWSS. Participants focused mostly on the need for micro-level economic incentives to encourage PrEP uptake. In addition, emphasis was placed on the need for more fundamental job training to help transgender women address their economic need and reliance on The Stroll economy.

The social environment of The Stroll was identified by all participants and key informants as generating HIV risk. Lower power to negotiate safe sex and HIV risk perceptions linked to sex work made PrEP, in theory, a highly desirable harm reduction strategy in participant narratives. However, the broader social context raised a number of significant barriers to translating acceptance of PrEP’s utility in preventing HIV into broader acceptance and uptake. Medical, as well as broader institutional mistrust, emerged as an important social determinant relevant to PrEP acceptance among transgender women, and was touched upon by both participants and key informants. Medical mistrust is deeply rooted in historical experiences of abuse, neglect, and mistreatment of minorities by public health services and health care providers [42, 43]. In this study context, medical mistrust and belief that PrEP might be harmful must be considered in light of the intersection of historical abuse of Black and transgender communities by Johns Hopkins Hospital [42, 43], as well as by other institutions including the police [8, 38]. Taking account of the fact that most participants in this study reported good current health provider relations in a context of gender affirming medical providers, further research is needed to understand how medical mistrust may function as a barrier to PrEP uptake.

Participant narratives suggested that transgender women’s attitudes and views around health seeking are not homogeneous and vary over time, based on factors such as increased access to positive gender affirming healthcare experiences and wider socially and structurally affirming experiences (e.g., fewer negative police interactions, stable housing) which may mitigate historically rooted medical mistrust. For TWSS who might be least likely to be accessing healthcare and are having current abusive experiences with city institutions (e.g., police, shelters, neighborhood associations), historical medical mistrust may have a stronger influence and warrants further research in this context. Consistent with broader literature [31], co-locating PrEP with gender affirming care services was identified in key informant interviews as an important way to take account of trans women’s primary health concerns (eg, gender-affirmative care and management of gender transition) while building acceptance and trust around the delivery of HIV prevention services.

In-depth interview participants and stakeholders reported the significance of PrEP related stigma in creating barriers to acceptance and uptake. The city PrEP campaign targeting the LGBTQ community, in particular men who have sex with men, represented a clear example of a public health campaign that reinforced PrEP stigma in separating out the LGBTQ community as “high risk” for HIV, rather than adopting a more inclusive marketing strategy that portrayed PrEP as for everyone. In addition, and equally prevalent in participant narratives, was the belief that many within the transgender and wider community conflated PrEP with HIV treatment, with HIV related stigma still pervasive in this study setting. An additional stigma experienced by nearly all participants is felt stigma associated with engaging in sex work. The internalization of stigmatizing attitudes can infiltrate and undermine an individuals’ internal perception of themselves, with consequences for their ability to function on a social and/or occupational level [12].

Our findings point to the importance of these intersecting stigmas on health seeking and engagement in PrEP for TWSS. In particular the convergence of PrEP related stigma, which is fundamentally sexual stigma [16] with sex worker related stigma, rooted in perceptions of immoral sexual behaviors and HIV risk, could amplify the stigma that impedes PrEP acceptance and uptake among TWSS. These findings point to the importance of PrEP interventions that address the particular challenges stigma represents to acceptance and uptake of PrEP among TWSS [27]. Participants in particular spoke of the importance of marketing PrEP for everyone, while simultaneously using positive social spaces specific to the transgender community to increase awareness and acceptance, namely the ball scene. In line with previous research in LA House and Ball communities [18] any attempts to engage with the house and ball community leaders in Baltimore must be done in a meaningful way based on trust and mutual collaboration with researchers in the design of effective and population sensitive HIV prevention agendas.

In terms of the physical environment of The Stroll, policing and violence emerged as two additional factors in the lives of TWSS that participants spoke about as indirectly impacting acceptance and uptake of PrEP. It was clear that extreme enacted violence on or near The Stroll, including the murder of trans women, had a deleterious impact on some trans women’s mental health and interest in self-care and health seeking. The most direct impact of policing on PrEP delivery, identified in a number of stakeholder interviews, was the harassment by the police of the only TWSS Stroll outreach operating at the time in this study setting. In addition, one stakeholder had heard reports of Truvada being confiscated by the police, but this was not verified in any other interviews. The indirect and direct impacts of policing on TWSS health seeking behaviors warrants more research, but in this setting, it is clear that for HIV prevention strategies to be effective, local health and harm reduction providers need to find a way to partner with police and reframe approaches to policing that focus on creating an enabling environment for harm reduction, including delivery of HIV prevention messaging and services.

## Limitations

This study has a number of limitations. The sample of TWSS all reported sex work as part of the inclusion criteria for the parent cohort study, SAPPHIRE. However, when interviewed, a few of the older women reported that they were no longer actively engaged in selling sex on The Stroll. Nonetheless, all interviewees were able to provide illuminating accounts from recent lived experience on The Stroll and the current conditions for TWSS. Transgender women in this study setting have been subject to a high level of research and there has been concern expressed by transgender community-based organizations around research fatigue, and what benefit repeat observational research has for the community. Therefore, it is possible that our data excluded the views of those in the community who were feeling this research fatigue.

## Conclusion

This study provides unique insights into challenges TWSS face in initiating PrEP and underscores the importance of developing interventions that account for the structural macro and micro risk environment. Our study results go beyond previous studies findings that focused on environmental level risk factors and PrEP delivery to transgender women as we examined the multi-level risk environment of The Stroll on PrEP delivery to TWSS. The emphasis on the role of sex work as an additional factor exacerbating transgender women’s existing structural vulnerability to poor health and well-being adds a critical perspective. Structural vulnerability in the form of gender, racial, economic and social discrimination among Black and Latina transgender women has been shown to create barriers to health seeking and uptake of harm reduction strategies [15, 19, 36]. Here we draw attention to the mutually reinforcing impact of the risk environment of The Stroll and call for increased social science perspectives in the design of PrEP interventions and delivery that attend to the complexity and syndemics of the social and structural contexts in which HIV prevention care is enacted and experienced by TWSS.

## Data Availability

Given the sensitive nature of topics covered in our in-depth interviews and the potential risk of identifiability to participants, the interview transcripts comprising the dataset will not be made available to those outside the study team without a fully executed data use agreement. Interested parties should contact the corresponding author for access to the data.
